# Global Run-On sequencing to measure nascent transcription in *C. elegans*

**DOI:** 10.1016/j.xpro.2021.100991

**Published:** 2021-12-04

**Authors:** Piergiuseppe Quarato, Germano Cecere

**Affiliations:** 1 Institut Pasteur, Mechanisms of Epigenetic Inheritance, Department of Developmental and Stem Cell Biology, UMR3738, CNRS, 75724 Cedex 15 Paris, France

**Keywords:** Cell separation/fractionation, Gene Expression, Genomics, Model Organisms, Molecular Biology, RNAseq, Sequencing

## Abstract

Global Run-On sequencing (GRO-seq) is one of the most sensitive techniques to detect nascent transcription from RNA polymerase (Pol) at a genome-wide level. The protocol incorporates labeled ribonucleotides into nascent RNAs from Pol I, II, and III. We have adapted the GRO-seq protocol to the nematode *Caenorhabditis elegans* to measure transcription from embryos and adult worms. Here, we provide a detailed overview of the protocol highlighting the critical steps for generating successful libraries.

For complete details on the use and execution of this protocol, please refer to Quarato et al. (2021).

## Highlights

• Isolation of nuclei from *C. elegans* adults or embryos
• Labeling of nascent transcripts with a biotinylated nucleotide
• Three immunoprecipitation steps allow the efficient removal of the background
• The library preparation strategy allows further removal of the background

## Graphical abstract

**Figure:**
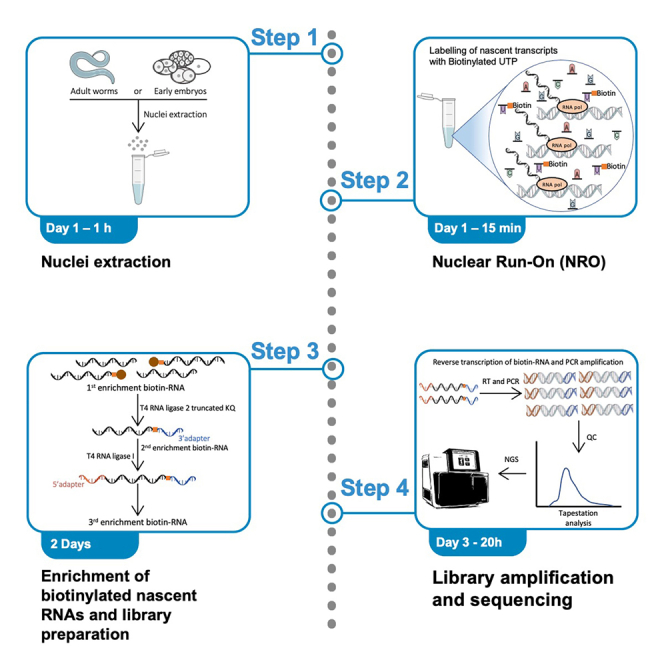

## Before you begin

### Overview of the protocol

GRO-seq is a powerful technique to map RNA polymerase transcription at the genome-wide level. The assay has been initially developed for human cells (Core et al., 2008) and has been updated and adapted to work in fission yeasts, *Drosophila melanogaster,* and plant cells (Mahat et al., 2016). Here we describe the specific steps for producing GRO-seq libraries from *Caenorhabditis elegans* worms and embryos. We have used this protocol to generate GRO-seq libraries from as low as 1,000 worms or 40,000 embryos (Barucci et al., 2020; Quarato et al., 2021; Singh et al., 2021).

The protocol can be divided into three main steps:

1. Nuclei isolation and nuclear run-on with biotinylated-UTP
2. Enrichment of biotin-labeled nascent RNAs and library preparation
3. Library amplification and sequencing

### Collection of synchronized worms and early embryos

To grow synchronous populations of worms, collect embryos from gravid adults by bleaching and allow hatching for 12–20 h at 20°C to obtain synchronized L1 larvae. Seed 40,000 L1 larvae onto one 150 mm NGM plate and harvest worms at the desired developmental stage. Perform three washes with M9 buffer, remove the supernatant, and flash-freeze in dry ice. Worms can be stored at −80°C for up to a year.

To collect populations of early embryos, grow four 150 mm NGM plates of synchronous worms until adulthood. Carefully monitor worms using a stereomicroscope and perform bleaching shortly after they start to produce the first embryos. Immediately after bleaching, perform three washes with a cold M9 buffer, remove the supernatant and immediately freeze in dry ice to block cell divisions. Embryos can be stored at −80°C for up to a year.

***Note:*** we seed 150 mm NGM plates with a layer of concentrated *Escherichia Coli* culture (100**×**). For information about NGM plates and M9 buffer composition, please refer to the WormBook (http://www.wormbook.org/).

**CRITICAL:** the first embryonic divisions happen in a relatively short time. Therefore, handling time should be minimized to avoid the collection of embryos at more advanced developmental stages. Always use cold reagents after bleaching to slow down embryonic cell divisions.

***Optional:*** If needed, embryos can be stained with DAPI and visualized using a fluorescent microscope to assess the developmental stage of the population. For more information about this step, please refer to (Quarato et al., 2021).

***Note:*** In principle, the protocol described for collecting early embryos can be adapted to obtain embryos at different developmental stages. Also, embryos already laid on the plate can be physically separated from gravid adults by filtration, and two populations of embryos, early- and late-embryos, can be collected. However, we have never performed GRO-seq on embryos older than the 100-cell stage.

## Key resources table

**Table:** 

REAGENT or RESOURCE	SOURCE	IDENTIFIER
**Chemicals, peptides, and recombinant proteins**		

CaCl_2_ (1 M)	Sigma-Aldrich	Cat#21115
MgCl_2_ (1 M)	Thermo Fisher Scientific	Cat#AM9530G
Tris-HCI Buffer, pH 7.5 (1 M)	Thermo Fisher Scientific	Cat#15567027
TERGITOL™ solution Type NP-40, 70% in H2O	Sigma-Aldrich	Cat#NP40S
Glycerol for molecular biology	Sigma-Aldrich	Cat#G5516
DTT (1 M)	Thermo Fisher Scientific	Cat#P2325
Tris-HCI Buffer, pH 8 (1 M)	Thermo Fisher Scientific	Cat#BP1758
EDTA (500 mM)	Thermo Fisher Scientific	Cat#15575
NaCl (5 M)	Thermo Fisher Scientific	Cat#AM9759
NaOH	Sigma-Aldrich	Cat#S5881
Triton X-100	Sigma-Aldrich	Cat#T8787
TWEEN® 20	Sigma-Aldrich	Cat#P9416
KCl (1 M)	Sigma-Aldrich	Cat#60142
Ribonucleotide Solution Set	New England Biolabs	Cat#N0450
DNase/RNase-Free Distilled Water	Thermo Fisher Scientific	Cat#15667708
N-Lauroylsarcosine sodium salt (sarkosyl)	Sigma-Aldrich	Cat#L5777
Biotin-11-UTP	Jena Biosciences	Cat#NU-821-BIOX
Halt™ Protease Inhibitor Cocktail, EDTA-free (100**×**)	Thermo Fisher Scientific	Cat#78425
RiboLock RNase Inhibitor (40 U/μL)	Thermo Fisher Scientific	Cat#EO0381
TRI Reagent™ Solution	Thermo Fisher Scientific	Cat#AM9738
TRIzol™ LS Reagent	Thermo Fisher Scientific	Cat#10296028
1-Bromo-3-chloropropane (BCP)	Sigma-Aldrich	Cat#B9673
GlycoBlue™ Coprecipitant	Thermo Fisher Scientific	Cat#AM9515
2-Propanol for molecular biology	Sigma-Aldrich	Cat#I9516
Ethanol, Absolute (200 Proof), Molecular Biology Grade	Fisher Scientific	Cat#BP2818100
Dynabeads™ MyOne™ Streptavidin C1	Thermo Fisher Scientific	Cat#65001
DynaMag™-2 Magnet	Thermo Fisher Scientific	Cat#12321D
T4 Polynucleotide Kinase	New England Biolabs	Cat#M0201
Phenol/Chloroform/Isoamyl Alcohol, 25:24:1 (v/v)	Sigma-Aldrich	Cat#516726
5PRIME Phase Lock Gel™ – Heavy	Quantabio	Cat#2302830
Sodium Acetate (3 M), pH 5.5, RNase-free	Thermo Fisher Scientific	Cat#AM9740
T4 RNA Ligase 2, truncated KQ	New England Biolabs	Cat#M0373
Agencourt RNAClean XP beads	Beckman Coulter	Cat#A63987
T4 RNA Ligase	Ambion	Cat#AM2141
SuperScript™ IV Reverse Transcriptase	Thermo Fisher Scientific	Cat#18090010
NEBNext® Ultra™ II Q5® Master Mix	New England Biolabs	Cat#M0544
Agencourt AMPure XP beads	Beckman Coulter	Cat#A63880

**Critical commercial assays**		

High Sensitivity D1000 ScreenTape	Agilent	Cat#5067-5584
High Sensitivity D1000 Reagents	Agilent	Cat#5067-5585
Qubit™ dsDNA HS Assay Kit	Thermo Fisher Scientific	Cat#Q32851
NextSeq 500/550 High Output Kit v2.5 (75 Cycles)	Illumina	Cat#20024906

**Oligonucleotides**		

3’ end adapter: 3SR adapter: /5rApp/NNNN TGGAATTCTCGGGTGCCAAGG/3ddC/	IDT	N/A
5’ end adapter: 5SR adapter: rGrUrUrCrArGr ArGrUrUrCrUrArCrArGrUrCrCrGrArCrGrArUr CrNrNrNrN	IDT	N/A
Reverse transcription primer: SR_RT : GCCTT GGCACCCGAGAATTCCA	IDT	N/A
PCR barcoded primers	IDT	See Table 1

**Software and algorithms**		

bcl2fastq	https://support.illumina.com/sequencing/sequencing_software/bcl2fastq-conversion-software.html	N/A
Cutadapt	https://cutadapt.readthedocs.io/en/stable/index.html	N/A
bowtie2	http://bowtie-bio.sourceforge.net/bowtie2/index.shtml	N/A
featureCounts	https://sourceforge.net/projects/subread/	N/A
DESeq2	https://bioconductor.org/packages/release/bioc/html/DESeq2.html	N/A
Samtools	http://www.htslib.org/	N/A
bamCoverage	https://deeptools.readthedocs.io/en/develop/content/tools/bamCoverage.html	N/A
Integrative Genomic Viewer (IGV)	https://software.broadinstitute.org/software/igv/download	N/A

**Other**		

WHEATON® DOUNCE DURA-GRIND™Stainlesss Steel Tissue Grinder, 7 mL	DWK Life Sciences	Cat#357572
DNA LoBind® Tubes	Eppendorf	Cat#0030108051
ART™ Wide Bore Filtered Pipette Tips	Thermo Fisher Scientific	Cat#2069G
ThermoMixer®	Eppendorf	Cat#5382000015
ThermoTop®	Eppendorf	Cat#5308000003

## Materials and equipment

***Note:*** we suggest purchasing molecular biology grade reagents for the preparation of the following buffers. However, it is possible to prepare in the lab most of the solutions needed. If so, solutions should be prepared with nuclease-free water and filter-sterilized with a 0.22 μm filter.

**Table:** Nuclei extraction buffer

Reagent	Final concentration	Amount
CaCl_2_ (1 M)	3 mM	150 μL
MgCl_2_ (1 M)	2 mM	100 μL
Tris-HCI Buffer, pH 7.5 (1 M)	10 mM	500 μL
NP-40 (10%)	0.25%	1.25 mL
Glycerol (50%)	10%	10 mL
DTT (1 M)	0.5 mM	25 μL
RNase inhibitor (40 U/μL)	4 U/mL	5 μL
Protease inhibitors (100**×**)	1**×**	500 μL
ddH_2_O	n/a	38 mL
**Total**	**n/a**	**50 mL**

Buffer without DTT, RNase inhibitor, and protease inhibitors can be stored at 4°C for up to 3 months.

**Table:** Freezing buffer NO-Glycerol

Reagent	Final concentration	Amount
Tris-HCI Buffer, pH 8 (1 M)	50 mM	2.5 mL
MgCl_2_ (1 M)	5 mM	250 μL
EDTA (500 mM)	0.1 mM	10 μL
ddH_2_O	n/a	47.24 mL
**Total**	**n/a**	**50 mL**

Buffer can be stored at 4°C for up to 3 months.

**Table:** Freezing buffer

Reagent	Final concentration	Amount
Tris-HCI Buffer, pH 8 (1 M)	50 mM	2.5 mL
Glycerol (50 %)	40 %	40 mL
MgCl_2_ (1 M)	5 mM	250 μL
EDTA (500 mM)	0.1 mM	10 μL
ddH_2_O	n/a	7.24 mL
**Total**	**n/a**	**50 mL**

Buffer can be stored at 4°C for up to 3 months.

**Table:** NRO 2**×** mix

Reagent	Final concentration	Amount
Tris-HCl Buffer, pH 8 (1 M)	12.5 mM	1 μL
MgCl_2_ (1 M)	6.25 mM	0.5 μL
DTT (100 mM)	1.25 mM	1 μL
KCl (1 M)	375 mM	30 μL
Sarkosyl (5%)	1.25%	20 μL
ATP (100 mM)	0.625 mM	0.5 μL
CTP (100 mM)	0.625 mM	0.5 μL
GTP (100 mM)	0.625 mM	0.5 μL
RNase inhibitor (40 U/mL)	1 U/mL	2 μL
ddH_2_O	n/a	24 mL
**Total**	**n/a**	**80 μL**

Prepare immediately before use and store it at room temperature (20°C–25˚C).

**CRITICAL:** Sarkosyl is associated with acute inhalation toxicity and can cause skin corrosion and serious eye damage. Handle with care.

**CRITICAL:** always keep the NRO 2**×** mix at room temperature (20°C–25˚C). Low temperatures would cause Sarkosyl to precipitate. Allow all reagents to equilibrate at room temperature before mixing. Prepare immediately before use and visually inspect the buffer before adding to the sample.

***Note:*** The table shows the volumes to perform one Nuclear Run-On (NRO) reaction. Scale-up accordingly if you are performing an NRO reaction on multiple samples. NRO reaction is performed in 200 μL final volume: 100 μL nuclei suspension + 80 μL NRO 2**×** mix + 20 μL Bio-11-UTP.

**Table:** Binding & Washing Buffer 2**×**

Reagent	Final concentration	Amount
Tris-HCl Buffer, pH 7.5 (1 M)	10 mM	50 μL
NaCl (5 M)	2 M	2 mL
EDTA (500 mM)	1 mM	10 μL
ddH_2_O	n/a	2.94 mL
**Total**	**n/a**	**5 mL**

Buffer can be stored at room temperature (20°C–25˚C) for up to 3 months.

***Optional:*** 0.04% Tween 20 can be added to the Binding & Washing Buffer 2**×** to reduce non-specific binding.

**Table:** Solution A

Reagent	Final concentration	Amount
NaOH (10 M)	100 mM	20 μL
NaCl (5 M)	50 mM	20 μL
ddH_2_O	n/a	1.96 mL
**Total**	**n/a**	**2 mL**

Prepare fresh every time you use it, keep at room temperature (20°C–25˚C), and use it over the day.

**CRITICAL:** NaOH can cause chemical burns. Handle with care and avoid any contact with skin and eyes.

***Optional:*** 0.02% Tween 20 can be added to Solution A to prevent beads to adhere to tube walls during washes.

**Table:** Solution B

Reagent	Final concentration	Amount
NaCl (5 M)	100 mM	40 μL
ddH_2_O	n/a	1.96 mL
**Total**	**n/a**	**2 mL**

Buffer can be stored at room temperature (20°C–25˚C) for up to 3 months.

***Optional:*** 0.02% Tween 20 can be added to Solution B to prevent beads to adhere to tube walls during washes.

**Table:** High Salt Wash buffer

Reagent	Final concentration	Amount
NaCl (5 M)	2 M	20 mL
Tris-HCl Buffer, pH 7.5 (1 M)	50 mM	2.5 mL
Triton X-100 (10%)	0.5%	2.5 mL
ddH_2_O	n/a	25 mL
**Total**	**n/a**	**50 mL**

Buffer can be stored at 4°C for up to 3 months.

**Table:** Medium Salt Wash buffer

Reagent	Final concentration	Amount
NaCl (5 M)	300 mM	3 mL
Tris-HCl Buffer, pH 7.5 (1 M)	10 mM	500 μL
Triton X-100 (10%)	0.1%	500 μL
ddH_2_O	n/a	46 mL
**Total**	**n/a**	**50 mL**

Buffer can be stored at 4°C for up to 3 months.

**Table:** Low Salt Wash buffer

Reagent	Final concentration	Amount
Tris-HCl Buffer, pH 7.5 (1 M)	5 mM	250 μL
Triton X-100 (10%)	0.1%	500 μL
ddH_2_O	n/a	49.25 mL
**Total**	**n/a**	**50 mL**

Buffer can be stored at 4°C for up to 3 months.

## Step-by-step method details

**CRITICAL:** all steps of the protocol are performed at 4°C unless stated differently. Pre-chill all reagents and equipment before use. Nuclease-free reagents need to be used in all steps of the protocol.

### Nuclei isolation

**Timing: 1 h**

The following steps describe the procedure to isolate intact nuclei from worms or embryos. The protocol has been tested with 1,000 to 40,000 worms and 40,000 to 300,000 early embryos.

1. Resuspend worms or embryos in 1.5 mL of cold Nuclei extraction buffer (NEB) and transfer to a pre-chilled stainless steel tissue grinder on ice.
2. Lyse worms to release nuclei by applying 40 dounce strokes.

**CRITICAL:** take a small aliquot of the lysate (10–20 μL) and check that the worms are lysed using a stereomicroscope. Intact bodies and embryos should be absent. If intact corpses are still present, increase the number of dounce strokes.

3. Transfer the lysate to a pre-chilled 1.5 mL microcentrifuge tube.

***Note:*** Multiple samples can be processed simultaneously. If so, keep lysate from step 3 on ice while lysing the other samples. In this case, wash the metal dounce well between each sample with milliQ water. After processing all the samples, proceed immediately to step 4.

4. Remove debris by centrifuging at 100×*g* for 4 min at 4°C.
5. Transfer the supernatant to a new 1.5 mL low binding microcentrifuge tube.

**CRITICAL:** carefully collect the supernatant without disturbing the debris pellet. If necessary, repeat steps 4 and 5 until lysate is clear from debris. A residual 50–100 μL can be left in the tube to avoid debris carryover.

6. Pellet nuclei by centrifuging at 1,000**×***g* for 4 min at 4°C.
7. Remove supernatant and wash nuclei with 1 mL NEB.

**CRITICAL:** wash nuclei by pipetting up and down several times using a wide-bore tip. Gently pipette nuclei to preserve their integrity.

8. Pellet nuclei by centrifuging at 1,000**×***g* for 4 min at 4°C.
9. Repeat steps 7 and 8 three more times for a total of 4 washes with NEB.
10. Wash nuclei in 1 mL Freezing buffer NO-Glycerol.

**CRITICAL:** wash nuclei by pipetting up and down several times using a wide-bore tip. Gently pipette nuclei to preserve their integrity.

11. Pellet nuclei by centrifuging at 1,000**×***g* for 4 min at 4°C and discard the supernatant.
12. Resuspend nuclei in 100 μL Freezing buffer.

**Pause point:** nuclei in the Freezing buffer can be stored at −80°C for up to 1 year.

### Nuclear run-on (NRO) reaction

**Timing: 15 min**

NRO is performed on isolated nuclei in the presence of Bio-11-UTP. Sarkosyl is added to prevent *de novo* assembly of the pre-initiation complex and avoid new transcriptional initiation. A negative NRO control reaction can be added at this step. In the negative NRO reaction, UTP is used instead of Biotinylated-UTP. If you are performing the experiment for the first time, the addition of negative control is strongly recommended.

13. Prepare the NRO 2x mix (the recipe is listed in the “Materials and equipment” section), and preheat it at 30°C before use.
14. Dispense 80 μL of the NRO 2**×** mix in a new low binding microcentrifuge tube.
15. Add 20 μL of Bio-11-UTP 10 mM to the NRO 2**×** mix (final Bio-11-UTP concentration: 1 mM) and gently mix.

**CRITICAL:** if running a negative NRO reaction, replace Bio-11-UTP with 0.5 μL of UTP 100 mM and 19.5 μL of nuclease-free water.

16. Add 100 μL of lysed nuclei from the previous step 12. Gently mix the reaction well using a wide bore tip.
17. Incubate the reaction for 3 min at 30°C.
18. Transfer the tubes on ice and add 50 μL ice-cold nuclease-free water and 750 μL TRIzol LS reagent and mix well to stop the reaction.
19. Incubate nuclei in TRIzol for 5 min at room temperature (20°C–25˚C) to permit complete dissociation of the nucleoprotein complexes.

**CRITICAL:** TRIzol has an acute oral and dermal toxicity. Always handle in a chemical hood. Contact with eyes and skin should be avoided.

**Pause point:** Nuclei in TRIzol can be stored at −80°C for up to a week (we have not tested longer storage).

### RNA isolation using TRIzol

**Timing: 1 h 15 min**

Total RNA, including biotinylated nascent RNA, is isolated using TRIzol.

20. Add 100 μL of 1-Bromo-3-chloropropane (BCP) and mix it well by vortexing.

**CRITICAL:** BCP is toxic upon inhalation. Always handle in a chemical hood.

21. Incubate at RT for 15 min.
22. Centrifuge at 12,000**×***g* for 15 min at 4°C, then transfer the aqueous phase (upper phase) containing the RNA to a new low binding microcentrifuge tube.

***Note:*** Avoid transferring any of the interphase or organic layer into the pipette when removing the aqueous phase. The tube can be angled at 45° to facilitate the process. Approximately 450 μL of aqueous phase can be collected at this step.

23. Add 2 μL of GlycoBlue and mix it well by vortexing for 5–10 s.
24. Add 500 μL of isopropanol and mix it vigorously by vortexing for 10–15 s.
25. Incubate at RT for 15 min to precipitate the RNA.
26. Centrifuge at 12,000**×***g* for 15 min at 4°C to pellet the RNA.
27. Remove the supernatant and add 1 mL of 75 % Ethanol to the RNA pellet.
28. Centrifuge at 7,500**×***g* for 5 min at 4°C.

***Note:*** After centrifugation, a blue pellet should be visible at the bottom of the tube.

**CRITICAL:** Carefully remove the supernatant without disturbing the RNA pellet.

29. Remove the supernatant and air dry the pellet for 1–2 min.
  ***Note:*** to completely remove the supernatant without disturbing the pellet you can use the following procedure:
  a. Remove the supernatant using a p1000 micropipette.
  b. Briefly centrifuge to collect all residual supernatant at the bottom of the tube. At this point, the pellet should be on the side of the tube.
  c. Using a p200 micropipette, gently push at the bottom-center of the tube and remove all residual ethanol. Always check that the pellet is not entering the tip.
  d. Briefly air dry the pellet.
    **CRITICAL:** do not over-dry the RNA pellet as this would make it difficult to resuspend it.

### RNA fragmentation

**Timing: 8 min**

30. Resuspend the RNA pellet from the previous step 29 in 8 μL water.

**Pause point:** RNA can be stored at −80°C for up to a week.

***Note:*** The analysis of RNA quality/integrity is not required at this step. This is because degradation fragments cannot be cloned with the library preparation strategy described in this protocol. Only RNA molecules with 3′-hydroxyl biotinylated ends will be suitable for cloning. In addition, the RNA quality control methods will primarily detect mature mRNAs and not Polymerase-bound nascent RNAs, which are much less abundant.

31. Add 2 μL of 5**×** reverse transcriptase buffer (provided with SuperScript™ IV Reverse Transcriptase) and mix it well by pipetting or vortexing. Spin down the samples using a benchtop centrifuge.

***Note:*** RNA fragmentation can be performed using different buffers, including commercially available RNA fragmentation reagents, based on MgCl_2_ or ZnCl_2_. If using a different buffer, check that the final library size is correct and adjust the fragmentation time accordingly.

32. Incubate the samples at 95°C for 7 min.

***Note:*** incubation can be performed in a ThermoMixer equipped with Thermo Top. Alternatively, the sample can be transferred to a PCR tube and incubated in a thermocycler with the lid temperature set at 105°C. During the fragmentation time, it is possible to start preparing the Dynabeads magnetic beads to isolate biotinylated molecules.

33. Immediately block fragmentation by shifting the samples on ice.
34. Add 50 μL of ice-cold nuclease-free water and keep on ice for at least 1 min before proceeding to the next step.

### Preparation of dynabeads MyOne streptavidin C1

**Timing: 10 min**

Dynabeads are washed following the manufacturer’s instructions before binding to biotinylated RNA.

**CRITICAL:** This step is crucial since the beads are not supplied in RNase-free solutions.

***Note:*** all the washing steps are performed at room temperature (20°C–25˚C) unless stated differently.

35. Resuspend the Dynabeads™ magne/tic beads in the vial (i.e., vortex for >30 s, or tilt and rotate for 5 min).
36. Transfer 30 μL of Dynabeads™ magnetic beads per sample to a low binding microcentrifuge tube.

***Note:*** 30 μL of beads are sufficient for a sample of 1,000 to 40,000 adult worms or 40,000 to 300,000 embryos. If performing the experiment with a different number of worms/embryos, the volume of beads should be scaled accordingly.

37. Add 1 mL of Binding & Washing Buffer 1**×** and resuspend by pipetting or gentle vortexing for 5–10 s. Then, briefly centrifuge the tube using a benchtop centrifuge.
38. Place the tube on a magnet for 1 mi/n or until the solution appears clear and discard the supernatant.
39. Remove the tube from the magnet and resuspend the magnetic beads in a Binding & Washing Buffer volume 1**×** equal to the initial volume of beads taken from the vial at step 36. Resuspend beads by pipetting or gentle vortexing for 5–10 s. Then, briefly centrifuge the tube using a benchtop centrifuge.
40. Place the tube on a magnetic stand for 1 min or until the solution appears clear and discard the supernatant.
41. Repeat steps 39–40 twice for a total of 3 washes.
42. Wash the beads twice in Solution A for 2 min. Use a volume of Solution A equal to or larger than the initial volume of Dynabeads™ magnetic beads initially taken from the vial.
43. Wash the beads once in Solution B. Use a volume of Solution B equal to the volume used for Solution A.
44. Resuspend the beads in 60 μL per sample of 2**×** Binding & Washing Buffer.

***Note:*** The washed beads can be prepared in advance and stored at 4°C for 1 day.

### 1^st^ enrichment of biotinylated RNA molecules

**Timing: 50 min**

Biotinylated nascent RNAs are enriched by binding to Dynabeads™ magnetic beads. After binding, the beads coated with the biotinylated molecules are washed using high salt buffers to efficiently remove non-biotinylated molecules.

45. Add 60 μL of washed beads from previous step 44 to 60 μL of fragmented RNA from previous step 34.
46. Incubate for 30 min at 25°C with 1200 rpm agitation (15’’ ON 45” OFF) in a ThermoMixer.
47. Briefly spin the tubes using a benchtop centrifuge and place on a magnetic rack for 1 min or until the solution appears clear.
48. Remove the supernatant and add 1 mL of High salt buffer. Incubate on rotation for 5 min at 4°C.
49. Briefly spin the tubes using a benchtop centrifuge and place on a magnet for 1 min or until the solution appears clear.
50. Remove the supernatant and add 1 mL of Medium salt buffer. Incubate on rotation for 5 min at 4°C.
51. Briefly spin the tubes using a benchtop centrifuge and place on a magnet for 1 min or until the solution appears clear.
52. Remove the supernatant and add 1 mL of Low salt buffer. Incubate on rotation for 5 min at 4°C.
53. Briefly spin the tubes using a benchtop centrifuge and place on a magnet for 1 min or until the solution appears clear.
54. Remove the supernatant, add 1mL TRI Reagent solution, and proceed to RNA isolation as previously described (Refer to “RNA isolation using TRIzol”, steps 20–29).

**Pause point:** Beads in TRIzol can be stored at −80°C for up to a week.

### RNA 5′ phosphorylation

**Timing: 35 min**

The RNA fragmentation step produces fragmented RNA molecules with 3′-phosphate and 5′-hydroxyl termini, not suitable for the adapter ligation steps, which require a 3′-hydroxyl and a 5′-phosphate terminus. Therefore, with the following steps, 5′-hydroxyl ends are phosphorylated using the Polynucleotide kinase.

***Note:*** Since incorporating Bio-11-UTP stall RNA polymerases, all nascent RNAs should terminate with a 3′-hydroxyl biotinylated end. Therefore, RNA molecules terminating with a 3′-phosphate end, result from the fragmentation steps and represent the background for this protocol. For this reason, 3′-phosphate ends do not need to be repaired to allow ligation of the adapter.

55. Resuspend RNA after TRIzol purification in 20 μL water.

**Pause point:** RNA can be stored at −80°C for up to a week.

56. Setup RNA 5′ phosphorylation reaction in 100 μL final volume by adding to the RNA from the previous step:
  a. 10 μL 10**×** Polynucleotide Kinase Buffer(
  b. 1 μL 100 mM ATP
  c. 0.5 μL 40 U/μL RNase inhibitor
  d. 2μL 100 U/μL T4 Polynucleotide Kinase
  e. 66.5 μL water.
57. Incubate the reaction at 37°C for 30 min.
58. Add 200 μL of ice-cold nuclease-free water, keep the sample on ice and proceed to the next step.

### RNA purification using Phenol:Chloroform

**Timing: 2 h**

59. Spin “5PRIME Phase Lock Gel™ – Heavy” tubes for 2 min at 12,000**×***g* at room temperature (20°C–25˚C) to pellet the gel.
60. Add the solution from the previous step 58 to a pre-spun Phase Lock tube.
61. Add 1 volume (300 μL) of Phenol/Chloroform/Isoamyl Alcohol and mix thoroughly by inverting 10–15 times.

**CRITICAL:** do not vortex the tube to avoid disturbing the Phase Lock gel.

**CRITICAL:** Phenol:Chloroform is toxic upon inhalation. Always handle in a chemical hood. Contact with eyes and skin should be avoided.

62. Centrifuge at 12,000**×***g* for 5 min at room temperature (20°C–25˚C) and transfer the aqueous phase containing the RNA (upper phase) to a fresh low binding microcentrifuge tube.
63. Add 30 μL of 3 M sodium acetate and 2 μL of GlycoBlue and vortex for 5–10 s.
64. Add 900 μL ethanol and vortex vigorously for 10–15 s.
65. Precipitate the RNA by incubating for 1h at −20°C.

**Pause point:** RNA can be precipitated overnight or over-weekend.

66. Centrifuge to pellet the RNA at 12,000**×***g* for 30 min at 4°C.
67. Remove the supernatant and add 1 mL of 75 % Ethanol to the RNA pellet.

***Note:*** After centrifugation, a blue pellet should be visible at the bottom of the tube.

**CRITICAL:** Carefully remove the supernatant without disturbing the RNA pellet.

68. Centrifuge at 12,000**×***g* for 5 min at 4°C.
69. Remove the supernatant and air dry the pellet for 1–2 min.

**CRITICAL:** do not over-dry the RNA pellet as this would make it difficult to resuspend it.

70. Resuspend the RNA in 5 μL water.

### 3′ adapter ligation

**Timing: 16 h**

Biotinylated RNA molecules are ligated to the 5′ pre-adenylated DNA adapter using an optimized version of the T4 RNA ligase. This strategy reduces the formation of ligation artifacts such as concatemers or circles.

71. Perform adapter ligation in 20 μL final volume by adding to the RNA from the previous step:
  a. 1 μL 10 μM 3SR adapter
  b. 2 μL 10**×** Ligation Buffer
  c. 2 μL “T4 RNA Ligase 2, truncated KQ”
  d. 10 μL 50% PEG8000

**CRITICAL:** PEG8000 is very viscous. Carefully pipette the right amount of PEG in the solution and mix well by carefully pipetting up and down at least 20 times or vortexing. Visually inspect that the solution is well mixed.

72. Incubate the reaction for 16 h at 16°C in a ThermoMixer equipped with a Thermo top.

### RNA purification using agencourt RNAClean XP beads

**Timing: 40 min**

SPRI (Solid Phase Reversible Immobilization) beads are used to purify the RNA from the ligation reaction. No size selection is performed at this step.

***Alternatives:*** equivalent SPRI beads can be used at this step instead of Agencourt RNAClean XP beads.

73. Add 3 volumes (60 μL) of isopropanol to the ligation reaction from the previous step.
74. Add 1.8 volumes (36 μL) of Agencourt RNAClean XP beads.

**CRITICAL:** beads are very viscous. Mix well before use and carefully pipette the right volume of beads.

75. Mix well by pipetting up and down at least 15 times or by gentle vortexing and incubate for 20 min on ice.

***Note:*** during this time, it is possible to start washing Dynabeads (steps 35–44) for the 2^nd^ enrichment of biotinylated RNA molecules.

76. Briefly spin and place the tube onto a magnetic tube rack for 5 min to separate the beads from the solution.
77. Slowly pipette the cleared solution from the tube and discard.
78. While on the magnetic rack, dispense 200 μL of 75% ethanol into the tube, incubate for 30 s and discard the ethanol.
79. Repeat the previous step for a total of 2 washes.
80. Remove the tube from the magnetic rack and briefly spin down residual ethanol using a benchtop centrifuge.
81. Place the tube onto a magnetic tube rack and remove residual ethanol.
82. Let the beads air dry for 5 min.

**CRITICAL:** Over-drying the sample may result in a lower recovery.

83. Remove tubes from the rack and elute purified RNA from the beads by adding 61 μL water.

**CRITICAL:** manually resuspend the beads by pipetting up and down several times. Expel the elution buffer down the side of the tube to ensure the entire bead mass comes into contact with the buffer. Incubate for 30 s before proceeding to the next step.

84. Place the tubes onto a magnetic tube rack for 1 min to separate the beads from the solution. Then, slowly collect 60 μL RNA solution and transfer to a fresh tube.

**CRITICAL:** slowly collect the solution while keeping the tubes onto the magnetic rack to avoid beads' carryover. 1 μL of excess water is added at step 83 is added to avoid beads' carryover.

**Pause point:** RNA can be stored at −80°C for up to a week.

### 2^nd^ enrichment of biotinylated RNA molecules

**Timing: 1 h**

85. Heat purified RNA at 65°C for 5 min to denature secondary structures and place on ice for 1 min.
86. Perform a 2^nd^ enrichment of biotinylated RNA molecules by repeating steps 45–54.

### 5′ adapter ligation

**Timing: 2 h**

87. Resuspend RNA after trizol purification in 15 μL water.
88. Perform 5′ end ligation in a total volume of 20 μL by adding to RNA from the previous step:
  a. 1 μL 5SR adapter 10 μM
  b. 2 μL Ligation buffer 10**×**
  c. 2 μL T4 RNA Ligase
89. Mix well and incubate the reaction at 25°C for 2 h.
90. Proceed to RNA purification using Agencourt RNAClean XP beads as described in steps 73–84.

### 3^rd^ enrichment of biotinylated RNA molecules

**Timing: 1 h**

91. Heat purified RNA (60 μL) at 65°C for 5 min to denature secondary structures and place on ice for 1 min.
92. Proceed to the 3^rd^ enrichment of biotinylated molecules by repeating steps 45–54.

### Reverse transcription

**Timing: 1 h 20 min**

Ligated RNA molecules are reverse transcribed to produce complementary DNA molecules suitable for library amplification.

93. Resuspend RNA after TRIzol purification in 11 μL of water.
94. Perform reverse transcription reaction in a final volume of 20 μL by adding to RNA from the previous step:
  a. 1 μL SR_RT primer 50 mM
  b. 1 μL dNTP mix 10 mM
  c. 4 μL SSIV Buffer 5**×**
  d. 1 μL DTT 100 mM
  e. 1 μL RNase inhibitor
  f. 1 μL SuperScript IV reverse transcriptase
95. Mix well and incubate the reaction at 50°C for 1 h in a thermal cycler with the lid temperature set at 85°C.
96. Inactivate the reaction by heating the sample at 80°C for 10 min.
97. Keep the sample at 4°C until ready to proceed to the next step.

**Pause point:** reverse-transcribed cDNA can be stored at −20°C for up to a month.

### Test PCR amplification of libraries

**Timing: 45 min**

After reverse transcription, libraries are amplified by PCR using a universal primer and a barcoded primer to allow sample multiplexing. Primer sequences are listed in Table 1.

**Table 1:** Primers for library amplification

Primer name	Primer sequence
SR universal primer	AATGATACGGCGACCACCGAGATCTACACGTTCAGAGTTCTA CAGTCCG∗A
SR barcode primer 1	CAAGCAGAAGACGGCATACGAGAT**CGTGAT**GTGACTGGAGTT CCTTGGCACCCGAGAATTCC∗A
SR barcode primer 2	CAAGCAGAAGACGGCATACGAGAT**ACATCG**GTGACTGGAGTT CCTTGGCACCCGAGAATTCC∗A
SR barcode primer 3	AAGCAGAAGACGGCATACGAGATG**CCTAA**GTGACTGGAGTT CCTTGGCACCCGAGAATTCC∗A
SR barcode primer 4	CAAGCAGAAGACGGCATACGAGAT**TGGTCA**GTGACTGGAGTT CCTTGGCACCCGAGAATTCC∗A
SR barcode primer 5	CAAGCAGAAGACGGCATACGAGAT**CACTGT**GTGACTGGAGTT CCTTGGCACCCGAGAATTCC∗A
SR barcode primer 6	CAAGCAGAAGACGGCATACGAGAT**ATTGGC**GTGACTGGAGTT CCTTGGCACCCGAGAATTCC∗A
SR barcode primer 7	CAAGCAGAAGACGGCATACGAGAT**GATCTG**GTGACTGGAGTT CCTTGGCACCCGAGAATTCC∗A
SR barcode primer 8	CAAGCAGAAGACGGCATACGAGAT**TCAAGT**GTGACTGGAGTT CCTTGGCACCCGAGAATTCC∗A
SR barcode primer 9	CAAGCAGAAGACGGCATACGAGAT**CTGATC**GTGACTGGAGTT CCTTGGCACCCGAGAATTCC∗A
SR barcode primer 10	CAAGCAGAAGACGGCATACGAGAT**AAGCTA**GTGACTGGAGTT CCTTGGCACCCGAGAATTCC∗A
SR barcode primer 11	CAAGCAGAAGACGGCATACGAGAT**GTAGCC**GTGACTGGAGTT CCTTGGCACCCGAGAATTCC∗A
SR barcode primer 12	CAAGCAGAAGACGGCATACGAGAT**TACAAG**GTGACTGGAGTT CCTTGGCACCCGAGAATTCC∗A

∗ = Phosphorothioate bond.

The number of amplification cycles required to amplify the libraries is influenced by many factors and needs to be determined experimentally for each sample (for example, very early embryos have lower transcription levels than late embryos). Therefore, we suggest performing a test PCR amplification of the libraries using ¼ of the reverse transcription reaction.

**Figure 1:**
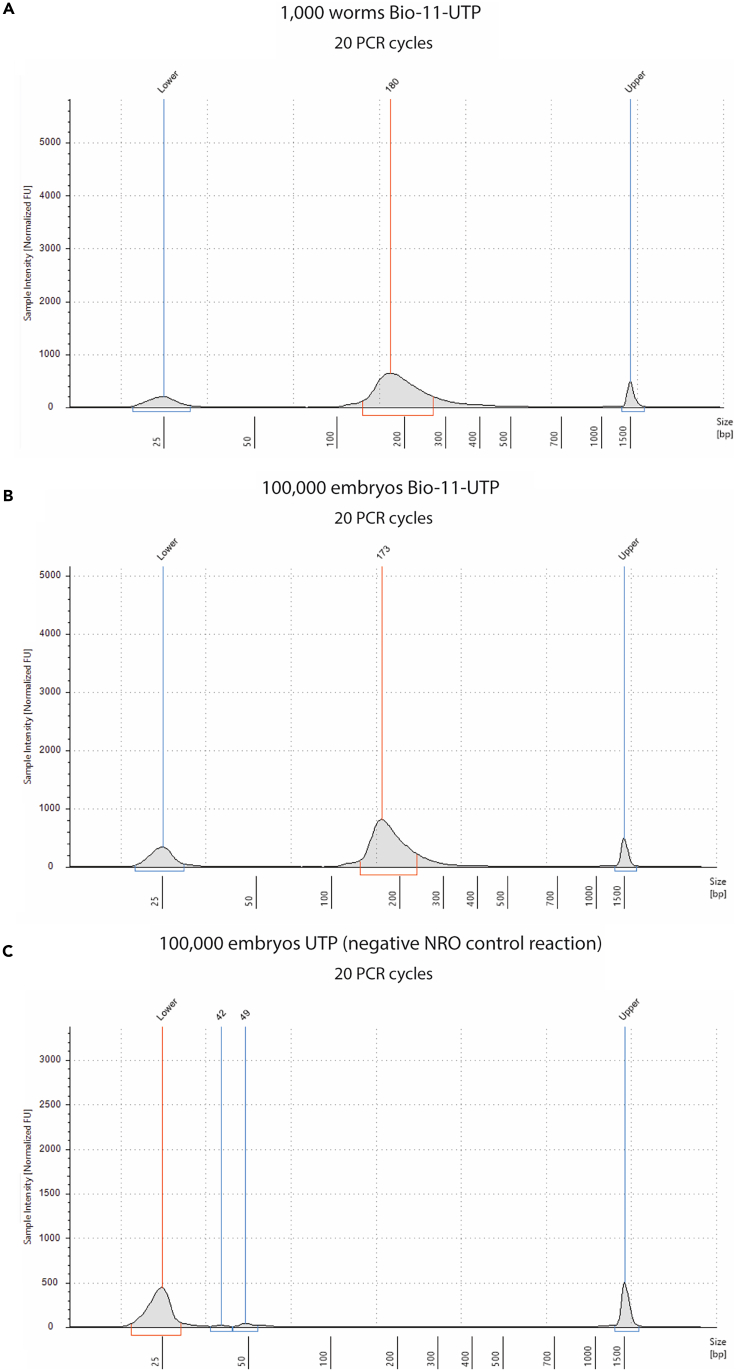
Tapestation analysis showing a proper library preparation result Example electropherograms from Tapestation analysis of GRO-seq libraries using 1,000 worms (A), 100,000 embryos (B), and 100,000 embryos after a negative NRO control reaction using UTP instead of Bio-11-UTP (C). No residual primers (< 100 bp) nor overamplification products (> 600 bp) are visible. The peak height is in the same range as the lower and upper markers for Figures (A and B). Number of PCR cycles used to amplify each library is indicated in the figure.

**CRITICAL:** The number of PCR cycles should be minimized to avoid overamplification, which increases PCR artifacts. Please refer to Figure 1 for an example of a correctly amplified library.

***Note:*** if multiple samples need to be sequenced together, use a different SR barcode primer per sample. The sequence of the SR barcode primers is available in Table 1.

98. Setup PCR reaction in a final volume of 50 μL by combining:
  a. 17 μL water
  b. 5 μL reverse-transcribed cDNA library
  c. 1.5 μL 10 μM SR universal primer
  d. 1.5 μL 10 μM SR barcode primer 10
  e. 25 μL NEBNext® Ultra™ II Q5® Master Mix 2**×**
99. Place the tube on a thermocycler with the heated lid set to 105°C and perform PCR amplification using the following PCR cycling conditions:

**Table:** 

PCR cycling conditions			
Steps	Temperature	Time	Cycles
Initial Denaturation	98°C	30 s	1
Denaturation	98°C	15 s	16–22 cycles∗
Annealing	61°C	30 s	
Extension	72°C	45 s	
Hold	4°C	Forever

***Note:*** We generally perform 20 PCR cycles for the test PCR for both adults and embryos.

### Purification of the PCR reaction using agencourt AMPure XP beads

**Timing: 30 min**

AMPure SPRI beads are used to remove salts, dNTP, primers, and primer dimers from the PCR reaction. 1.8 volumes of AMPure beads are used to recover fragments > 100 bp (expected size of amplicons 140–350 bp).

***Alternatives:*** equivalent SPRI beads can be used at this step instead of Agencourt AMPure XP beads. If different beads are used, the beads/sample ratio might vary. Refer to manufacturer guidelines to select the proper beads/sample ratio to purify fragments > 100 bp.

100. Add 90 μL AMPure XP beads to 50 μL PCR reaction.

**CRITICAL:** beads are very viscous. Mix well before use and carefully pipette the right volume of beads.

101. Mix well by pipetting up and down at least 15 times or by gentle vortexing and incubate for 5 min at RT.
102. Briefly spin using a benchtop centrifuge and place the tube onto a magnetic tube rack for 5 min to separate the beads from the solution.
103. Slowly pipette the cleared solution from the tube and discard.
104. While on the magnetic rack, dispense 200 μL of 75% ethanol into the tube, incubate for 30 s and remove the Ethanol.
105. Repeat the previous step for a total of 2 washes.
106. Remove the tube from the magnetic rack and briefly spin down residual ethanol using a benchtop centrifuge.
107. Place the tube onto a magnetic tube rack and remove residual ethanol.
108. Let the beads air fry for 5 min.

**CRITICAL:** Over-drying the sample may result in a lower recovery.

109. Remove the tube from the rack and elute purified DNA from the beads by adding 18 μL water.

**CRITICAL:** manually resuspend the beads by pipetting up and down several times. Expel the elution buffer down the side of the tube to ensure the entire bead mass comes into contact with the buffer. Incubate for 30 s before proceeding to the next step.

110. Place the tubes onto a magnetic tube rack for 1 min to separate the beads from the solution. Then, slowly collect 17 μL of cleaned DNA libraries and transfer them to a fresh tube.

**CRITICAL:** slowly pipette the solution while keeping the tubes onto the magnetic rack to avoid beads' carryover 1 μL of excess water is added at step 110 to prevent beads carryover.

### Quality control of purified DNA libraries and full-scale PCR amplification

**Timing: 10 min**

Purified DNA libraries are run on an Agilent Tapestation instrument using the High Sensitivity D1000 reagents to check for library size, purity, and amount.

111. Run 2 μL purified DNA libraries on an Agilent Tapestation instrument using the High Sensitivity D1000 reagents following manufacturer conditions.
112. Examine the result of the Tapestation run and determine the right PCR cycles for the full-scale PCR amplification. An example of Tapestation results for both worms and embryos can be found in the expected outcomes section and Figure 1.

**CRITICAL:** Adjust the PCR cycles of the final PCR amplification, calculating that each library should be in the same amount range of the upper and lower markers. Check for the presence of over-amplification products (high molecular size products) and reduce the number of PCR cycles to eliminate them (See expected outcomes, troubleshooting section, and Figure 3).

113. Setup a full-scale PCR reaction in a final volume of 50 μL by combining:
  a. 7 μL water
  b. 15 μL reverse-transcribed cDNA library (from step 97)
  c. 1.5 μL SR universal primer 10 μM
  d. 1.5 μL SR barcode primer 10 μM
  e. 25 μL NEBNext® Ultra™ II Q5® Master Mix 2**×**
114. Place the tube on a thermocycler with the heated lid set to 105°C and perform PCR amplification using the following PCR cycling conditions:

**Table:** 

PCR cycling conditions			
Steps	Temperature	Time	Cycles
Initial Denaturation	98°C	30 s	1
Denaturation	98°C	15 s	Determined at step 112
Annealing	61°C	30 s	
Extension	72°C	45 min	
Hold	4°C	forever

115. Purify the PCR reaction by repeating steps 100–110.
116. Run 2 μL of purified DNA libraries on an Agilent Tapestation instrument using the High Sensitivity D1000 reagents following manufacturer conditions.

**CRITICAL:** This step is crucial to assess the amount of each DNA library, the amplification status, and the absence of residual PCR primers before quantifying and sequencing. An example of TapeStation results for both worms and embryos can be found in the section expected outcomes and Figure 1.

### Quantification of DNA libraries and pooling for next-generation sequencing

**Timing: 20 min**

In the following steps, libraries are precisely quantified and combined in an equimolar ratio based on the size distribution and the concentration.

117. Quantify libraries using the Qubit™ dsDNA HS Assay Kit Always measure Qubit standard solutions before quantifying the DNA libraries.
  a. Prepare 200 μL of working solution for each sample by diluting the Qubit® dsDNA HS Reagent 1:200 in Qubit® dsDNA HS Buffer.
  b. To quantify the standard solutions transfer 190 μL working solution and 10 μL standard solution into a new Qubit 0.5 mL tube (for both standards #1 and #2).
  c. To quantify the libraries, transfer 198 μL of working solution and 2 μL of sample into a new Qubit 0.5 mL tube.
  d. Mix well, briefly spin using a benchtop centrifuge, and incubate for 2 min at RT.
  e. Read sample concentration by selecting the dsDNA High Sensitivity as the assay type on the Qubit instrument.
118. Combine the average peak size (step 116) and the concentration value to calculate the molarity of each library. Pool all libraries that have to be sequenced together in an equimolar ratio.

***Note:*** Library pooling can be performed by using the “Illumina Pooling Calculator” at https://support.illumina.com/help/pooling-calculator/pooling-calculator.htm

### High-throughput sequencing of GRO-seq libraries

**Timing: 12–24 h**

119. Sequence pooled GRO-seq libraries using an Illumina Sequencing platform compatible with the TruSeq approach.

***Note:*** we use a NextSeq-500 instrument and the NextSeq 500/550 High Output Kit v2.5 (75 Cycles) (Single-read sequencing). Generally, 20–30 million reads per sample provide enough coverage for both worms and embryos.

## Expected outcomes

After amplification, cDNA libraries are run on a Tapestation to check their quality and quantity. Expected library size range between 140–350 bp (Figures 1A and 1B). Residual PCR primers, primer-dimers, and empty products should be absent since they could negatively influence the library quantification and sequencing steps. The negative NRO control reaction should not show any library amplification product when amplified with the same number of PCR cycles as the biotin-UTP NRO reaction (Figure 1C).

## Quantification and statistical analysis

The steps below describe how to analyze GRO-seq raw data after sequencing to perform differential gene expression analysis and visualize data on the “integrative genomic viewer” (IGV).

1. Use bcl2fastq to demultiplex the samples and convert base call files (bcl) to fastq.gz files.

***Note:*** NextSeq 500 flowcell has 4 different lanes. Therefore, the result of the previous step will be 4 fastq.gz per sample. We usually concatenate all files corresponding to the same sample and work with a single file per sample. Files from different lanes can also be processed separately to assess differences between sequencing lanes.

2. Remove adapter sequence from raw reads using cutadapt or any equivalent software. Adapter sequence: TGGAATTCTCGGGTGCCAAGG
3. Remove random tetramers at both 5′ and 3′ ends using cutadapt or any equivalent software.

***Optional:*** random tetramers at both ends can also be used for removing PCR duplicates.

***Note:*** To improve the mapping step, we remove reads that are too short for mapping. Therefore, we apply a size selection criteria during raw reads processing, keeping only reads of 24 bp or longer after adapter trimmings. Thus, after random tetramers removal, reads will be 16 nucleotides or longer.

4. Map the trimmed reads on *C. elegans* genome using bowtie2.
5. Count features using featureCounts or any equivalent software. This step will produce a count table for the selected feature (for example, protein-coding genes, transposons, tRNA, rRNA, etc.).
6. Compute TPM or use deseq2 software to perform differential gene expression analysis.

**Figure 2:**
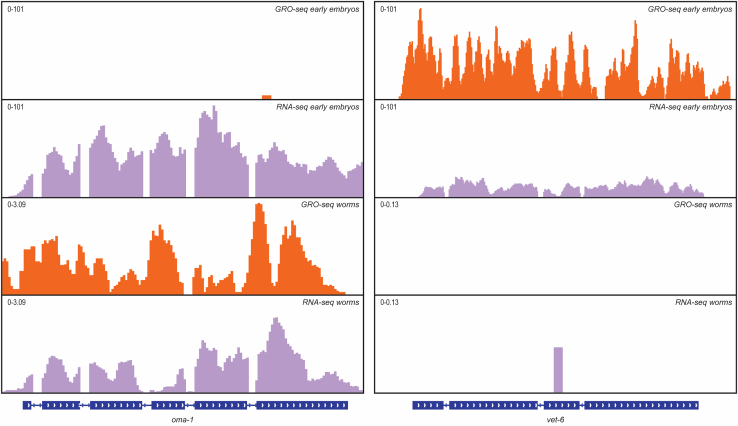
Genome browser example of a GRO-seq library compared to RNA-seq libraries A genomic view of two protein-coding genes showing normalized GRO-seq (orange) or RNA-seq (light purple) reads from early embryos or adults. oma-1 (left) is highly expressed in adult worms and is among the embryo's most abundant maternally inherited mRNAs. However, oma-1 is not transcribed in early embryos. vet-6 (right) is not expressed in adult worms, is not maternally inherited, and is among the earliest transcribed genes in embryos.

***Optional:*** to visualize genomic data, we suggest generating normalized bigwig files (Figure 2). This can be done by sorting and indexing bam files using samtools and converting them to normalized bigwig files using the bamCoverage package from deeptools.

## Limitations

Here, we presented a protocol to perform GRO-seq on as low as 1,000 L4/adult worms (around 2,000,000 nuclei) or 40,000 early embryos (around 800,000 nuclei). The input required for this protocol is much lower than the first reported GRO-seq in *C. elegans,* which required 100,000 worms (Cecere et al., 2014). Nonetheless, this minimum number of worms or embryos might still be difficult to achieve for specific samples, such as mutant strains with fertility defects. The main limitation of the protocol is the nuclei extraction procedure which has an estimated efficiency of 50%. Therefore, improvements in this step might help decrease the input material required to run GRO-seq in the future.

Another limitation is the high cost of the method. Indeed, reagents such as biotinylated nucleotides, streptavidin-conjugated beads, and ligases drastically influence the cost per sample. Home-production of the required enzymes and order of large batches of biotinylated nucleotides helps reduce the cost per sample of this protocol.

Ultimately, to understand the advantages and the limitations of the general GRO-seq procedure, we suggest referring to (Mahat et al., 2016).

## Troubleshooting

### Problem 1

No library product or > 25 PCR amplification cycles are required to obtain the libraries (step 112).

Possible causes:

Poor nuclei preparation quality.

Problems in the NRO reaction.

RNA degradation.

Low recovery in the SPRI purification steps.

### Potential solution

Potential solutions are listed below:

Stain nuclei with DAPI and check for their integrity by fluorescence microscopy. If nuclei are not intact, reduce the number of dounce strokes at step 2.

Check for precipitates in the NRO 2**×** mix. If present, prepare the solution again.

Always work on ice and use RNase-free reagents at all steps.

Be careful not to over-dry the beads after purification. Dry beads are difficult to resuspend and may result in low recovery of RNA or DNA.

### Problem 2

Libraries show the presence of unused primers or primer-dimers (< 100 bp) after Tapestation analysis (step 116).

### Potential solution

If primers are present (< 100 bp), perform a new round of library size selection using AMPure SPRI beads and perform a new Tapestation analysis.

***Note:*** We do not recommend performing gel purification to remove primer-dimers as this would result in sample loss. In our experience, an additional round of SPRI beads purification is sufficient to remove unused primers or primer-dimers and to obtain libraries of high quality.

### Problem 3

Libraries are overamplified and show peaks corresponding to high molecular weight products (> 600 bp) (step 112).

### Potential solution

**Figure 3:**
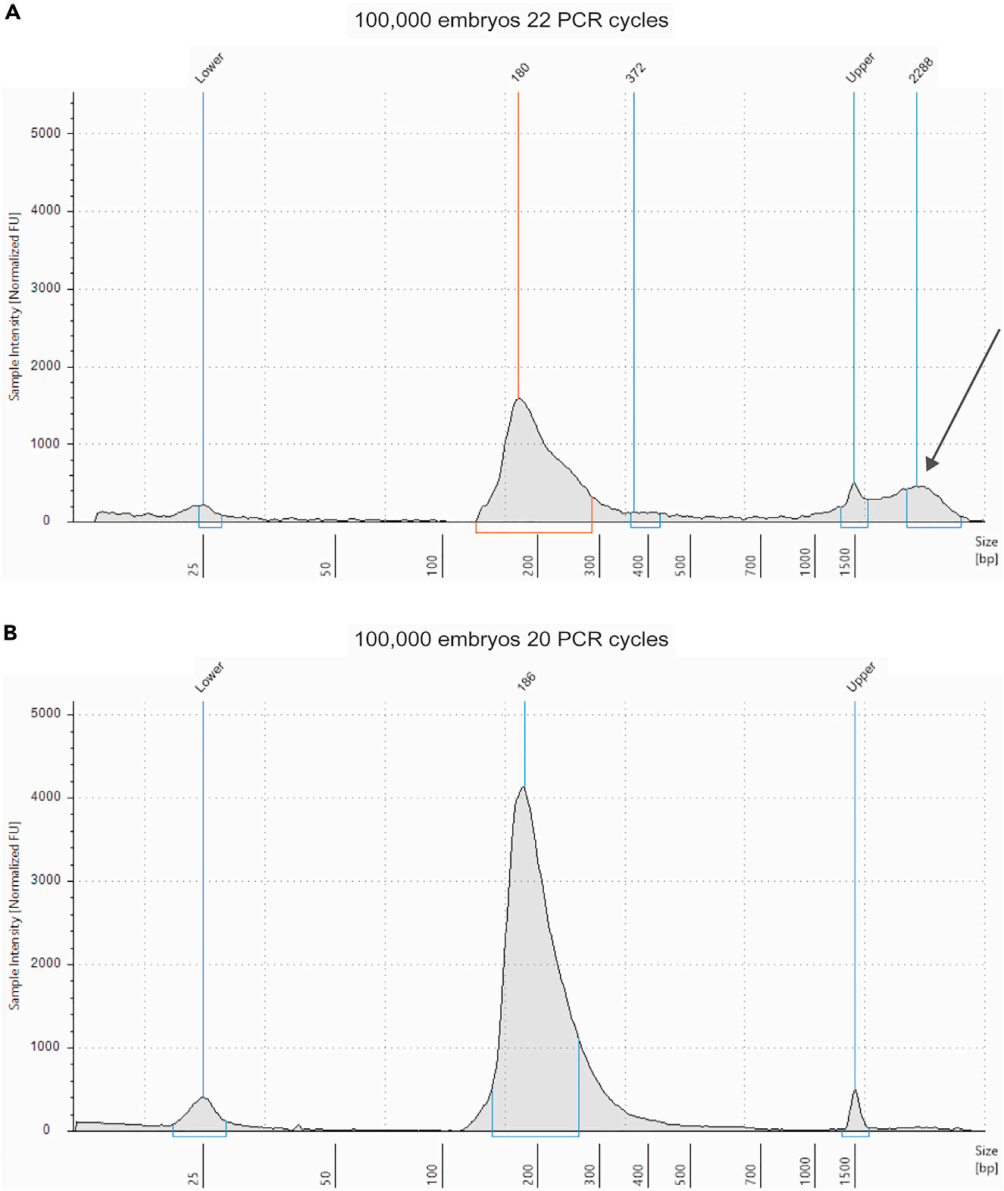
Tapestation analysis of an overamplified library (A) GRO-seq library from 100,000 embryos amplified with 22 PCR cycles. The electropherogram shows the presence of high molecular weight products (arrow). (B) Same library show in (A). amplified with 2 PCR cycles less. PCR artifacts are not present, and the size distribution of the library is correct.

Reduce PCR amplification by 2–4 cycles and perform a new Tapestation analysis. Figure 3 shows an example of the same library amplified with different PCR cycles.

### Problem 4

Streptavidin magnetic beads adhere to the tube wall after washing with solution A (step 42).

### Potential solution

The addition of 0.02% tween to Solution A can help to prevent this issue. Also, the use of low binding microcentrifuge tubes is suggested.

Furthermore, we have noticed that solution A might form precipitates few days after preparation. Therefore, we suggest preparing a new batch of solution A for each experiment.

### Problem 5

Small or no pellet recovered after TRIzol or Phenol:Chloroform purification (steps 26 or 66).

### Potential solution

The absence of pellet after RNA precipitation might be due to incomplete mixing of the aqueous phase containing the RNA with GlycoBlue and Isopropanol/Ethanol (steps 23–24 for TRIzol purification and 64–65 for Phenol:Chloroform purification).

Mix the sample again by vortexing at maximum speed for 15 s and repeat the precipitation and centrifugation steps.

If the pellet is still not visible, add 2 μL of GlycoBlue, mix thoroughly, and repeat the precipitation and centrifugation steps by increasing the centrifugation speed to 16,000**×***g*.

## Resource availability

### Lead contact

Further information and requests for resources and reagents should be directed to and fulfilled by the lead contact, Germano Cecere (germano.cecere@pasteur.fr).

### Materials availability

All materials are available commercially. A list of manufacturers for all reagents is provided in the key resources table section.

## Data Availability

Data are available upon reasonable request.

## References

[bib1] Barucci G., Cornes E., Singh M., Li B., Ugolini M., Samolygo A., Didier C., Dingli F., Loew D., Quarato P. (2020). Small-RNA-mediated transgenerational silencing of histone genes impairs fertility in piRNA mutants. Nat. Cell Biol..

[bib2] Cecere G., Hoersch S., O’keeffe S., Sachidanandam R., Grishok A. (2014). Global effects of the CSR-1 RNA interference pathway on the transcriptional landscape. Nat. Struct. Mol. Biol..

[bib3] Core L.J., Waterfall J.J., Lis J.T. (2008). Nascent RNA sequencing reveals widespread pausing and divergent initiation at human promoters. Science.

[bib4] Mahat D.B., Kwak H., Booth G.T., Jonkers I.H., Danko C.G., Patel R.K., Waters C.T., Munson K., Core L.J., Lis J.T. (2016). Base-pair-resolution genome-wide mapping of active RNA polymerases using precision nuclear run-on (PRO-seq). Nat. Protoc..

[bib5] Quarato P., Singh M., Cornes E., Li B., Bourdon L., Mueller F., Didier C., Cecere G. (2021). Germline inherited small RNAs facilitate the clearance of untranslated maternal mRNAs in C. elegans embryos. Nat. Commun..

[bib6] Singh M., Cornes E., Li B., Quarato P., Bourdon L., Dingli F., Loew D., Procaccia S., Cecere G. (2021). Translation and codon usage regulate Argonaute slicer activity to trigger small RNA biogenesis. Nat. Commun..

